# Down‐regulation of insulin‐like growth factor binding protein 5 is involved in intervertebral disc degeneration via the ERK signalling pathway

**DOI:** 10.1111/jcmm.14525

**Published:** 2019-07-10

**Authors:** Zhonghui Chen, Weibing Zhang, Nu Zhang, Yan Zhou, Geliang Hu, Mingdi Xue, Junhua Liu, Yaming Li

**Affiliations:** ^1^ Orthopedic Surgery Renmin Hospital of Wuhan University, Hubei General Hospital Wuchang District, Wuhan China; ^2^ Orthopedic Surgery Chibi Third Renmin Hospital Chibi China

**Keywords:** ERK, IGFBP5, intervertebral disc degeneration, nucleus pulposus

## Abstract

It is obvious that epigenetic processes influence the evolution of intervertebral disc degeneration (IDD). However, its molecular mechanisms are poorly understood. Therefore, we tested the hypothesis that IGFBP5, a potential regulator of IDD, modulates IDD via the ERK signalling pathway. We showed that IGFBP5 mRNA was significantly down‐regulated in degenerative nucleus pulposus (NP) tissues. IGFBP5 was shown to significantly promote NP cell proliferation and inhibit apoptosis in vitro, which was confirmed by MTT, flow cytometry and colony formation assays. Furthermore, IGFBP5 was shown to exert its effects by inhibiting the ERK signalling pathway. The effects induced by IGFBP5 overexpression on NP cells were similar to those induced by treatment with an ERK pathway inhibitor (PD98059). Moreover, qRT‐PCR and Western blot analyses were performed to examine the levels of apoptosis‐related factors, including Bax, caspase‐3 and Bcl2. The silencing of IGFBP5 up‐regulated the levels of Bax and caspase‐3 and down‐regulated the level of Bcl2, thereby contributing to the development of human IDD. Furthermore, these results were confirmed in vivo using an IDD rat model, which showed that the induction of Igfbp5 mRNA expression abrogated the effects of IGFBP5 silencing on intervertebral discs. Overall, our findings elucidate the role of IGFBP5 in the pathogenesis of IDD and provide a potential novel therapeutic target for IDD.

## 1 INTRODUCTION

Intervertebral disc degeneration (IDD) is a disease of discs that connect adjoining vertebrae, in which structural damage causes the degeneration of the disc and the surrounding area. This disease is a common cause of back pain in patients who do not achieve improvement with conservative management that possibly requires surgery.^1^, ^2^ It has been reported that the central features of IDD are the reduction of the nucleus pulposus (NP) cell population and the loss of extracellular matrix molecules (ECM), which eventually leads to major changes in the architecture and properties of the disc.^3^ The expression of several growth factors and their respective receptors has been reported in areas of degeneration in human and animal intervertebral discs.^4^ Thus, a comprehensive understanding of growth factors is essential to maximize the opportunities to develop therapeutic interventions that retard or reverse IDD.

There are several classical mitogens, such as platelet‐derived growth factor (PDGF), basic fibroblast growth factor (bFGF) and insulin‐like growth factor I (IGF‐I). IGF‐I and other members of the insulin family of proteins, including the insulin‐like growth factor binding proteins (IGFBPs), have been reported to concomitantly influence both metabolic and proliferative processes. IGFBP5 is one of the conserved IGFBPs that can translocate into the nucleus, as determined by the presence of a nuclear localization sequence.^5^ IGFBP5 has been shown to enhance cell growth and the remodelling and repair of bone. Daily subcutaneous injections of recombinant IGFBP5 protein stimulate osteoblast activity and bone accretion in ovariectomized mice.^6^ Moreover, a previous study demonstrated that IGFBP5 plays a crucial role in the carcinogenesis and progression of several types of cancer, such as oral and breast cancer, and its expression is usually dysregulated.^7^, ^8^, ^9^ However, the effect of IGFBP5 on the regulation of proliferative and apoptotic activities in NP cells is not well defined, even though IGFBP5 is expressed in NP cells.

Extracellular signal‐regulated kinase (ERK) is involved in an extremely conserved signalling pathway, known as the Ras‐Raf‐MEK‐ERK pathway, that is crucially involved in cell proliferation and survival and diverse cellular processes.^10^, ^11^ PD98059 is a highly selective in vitro inhibitor of MEK1 activation and the ERK cascade.^12^ A previous study demonstrated that exogenous and autocrine growth factors stimulate human intervertebral disc cell proliferation via the ERK and Akt pathways.^13^ However, the correlations between IGFBP5, the ERK signalling pathway, and IDD have not been elucidated. Therefore, we aimed to determine the role of IGFBP5 in NP cell proliferation and apoptosis and the involvement of the ERK signalling pathway in this process.

## 2 MATERIAL AND METHODS

### 2.1 Patient samples

Between February 2015 and March 2017, a total of 129 lumbar NP specimens were obtained from 129 patients with degenerative disc disease that underwent discectomy (mean age: 54.7 ± 9.6; range: 44‐69 years). Of these, 10 NP specimens were randomly selected for mRNA Solexa sequencing. The surgical indications were as follows: (a) failed conservative treatment and (b) progressive neurologic deficits such as progressive motor weakness or cauda equine syndrome. Patients with isthmic or degenerative spondylolisthesis, lumbar stenosis, ankylosing spondylitis, or diffuse idiopathic skeletal hyperostosis were excluded. The control samples were taken from 112 patients with fresh traumatic lumbar fracture (mean age: 21.1 ± 3.4, range: 18‐23 years) who underwent anterior decompressive surgery due to neurological deficits. Of these, 10 NP specimens were randomly selected for mRNA Solexa sequencing. To reduce the differences between the individuals and to obtain more data, an experimental design that used 10 (pathological) vs. 10 (control) specimens was thus employed for the mRNA Solexa sequencing in our study.

Routine MRI scans of the lumbar spine were taken for each patient before surgery, and the degree of disc degeneration was graded based on the T2‐weighted images using the Pfirrmann classification.^14^ According to the modified classification system used by the International Society for the Study of the Lumbar Spine,^15^ 101 samples showed protrusions, 21 showed sequestration and 7 showed subligamentous extrusions. The tissue specimens were first washed with phosphate‐buffered saline (PBS). Thirty‐eight of the 129 samples were obtained from the L4/L5 level, whereas 91 were from the L5/S1 level. The study protocol was approved by the ethics committee at our institution, and written informed consent was obtained from each participant.

### 2.2 Isolation and culture of human NP cells

Tissue specimens were first washed three times with PBS. The NP was then separated from the annulus fibrosus using a stereotaxic microscope and cut into pieces (2‐3 mm^3^). The samples were digested by 0.25 mg/mL type II collagenase (Invitrogen) for 12 hours at 37°C in Dulbecco's modified Eagle medium (DMEM; GIBCO) until the tissue blocks disappeared. After isolation, the NP cells were resuspended in DMEM containing 10% FBS (GIBCO), 100 mg/mL streptomycin, 100 U/mL penicillin, and 1% L‐glutamine and then incubated at 37°C in a humidified atmosphere with 95% air and 5% CO_2_. The confluent cells were detached by trypsinization and seeded into 35‐mm tissue culture dishes in complete culture medium (DMEM supplemented with 10% FBS, 100 mg/mL streptomycin and 100 U/mL penicillin) and incubated in a 5% CO_2_ environment at 37°C. The medium was changed every 2 days. The cells from the second passage were used for the subsequent experiments.

### 2.3 Cell transfections

The isolated cells were divided into the following groups: the blank group, the negative control (NC) group, the IGFBP5‐shRNA group, the IGFBP5 overexpression group, the ERK pathway inhibitor group (PD98059) and the IGFBP5‐shRNA + PD98059 group. The cells were seeded into 25‐cm^2^ culture flasks and allowed to reach 30%‐50% confluency. Serum‐free medium (SFM, 100 µL) was used to dilute 5 µL of Lipofectamine 2000 (Invitrogen) in sterile epoxy resin (EP) tubes, which were then incubated at room temperature for 5 minutes. For the IGFBP5‐shRNA group, short hairpin RNAs (shRNA) with sequences complementary to those of the target genes were subcloned into the pLKO.1 lentiviral vector (Addgene). Viral packaging was performed according to the manufacturer's protocol (Clontech Laboratories, Addgene). For the viral infections, NP cells were plated overnight and then infected with retroviruses or lentiviruses in the presence of polybrene (6 μg/mL; Sigma‐Aldrich) for 6 hours. After 48 hours, the infected cells were selected with different antibiotics. The target sequence for the IGFBP5 shRNA (IGFBP5sh) was 5′‐GCAGATCTGTGAATATGAA‐3′. For the NC group, a scrambled shRNA (Scramsh) was used as a control and was purchased from Addgene.

For the IGFBP5 overexpression group, IGFBP5 was expressed in vitro using the gateway vector pLenti6.2/V5‐DEST (Invitrogen), hereafter referred to as pLenti6.2‐IGFBP5. First, IGFBP5 was amplified from pCR4‐TOPO‐IGFBP5 (Thermo Scientific). According to the manufacturer's protocols, IGFBP5 was then shuttled into the pENTR/D‐TOPO vector (Invitrogen) and recombined into pLenti6.2/V5‐DEST (Invitrogen). In addition, to suppress ERK expression, the cells were treated with PD98059 according to the manufacturer's instructions.

### 2.4 RNA isolation

TRIzol (Invitrogen) and an RNeasy mini kit (Qiagen) were used for the extraction of the total RNA from NP cells according to the manufacturers’ protocols. Subsequently, RNA was eluted in 50 mL of nuclease‐free water and stored at −80°C prior to further analysis. The RNA concentration was measured using a NanoDrop ND‐1000 spectrophotometer (NanoDrop Technologies).

### 2.5 Transcriptome sequencing

The total RNA from 10 patients and 10 controls was purified directly for transcriptome sequencing analysis using the Illumina HiSeq 2500 platform according to the manufacturer's instructions. The library used for sequencing was generated using an Illumina TruSeq RNA Sample Preparation Kit (Illumina), which was used for RNA fragmentation, random hexamer‐primed first‐strand cDNA synthesis, dUTP‐based second‐strand cDNA synthesis, end repair, A‐tailing, adaptor ligation and library PCR amplification. The image files were generated by the sequencer and processed to produce digital‐quality data. After masking the adaptor sequences and removing the contaminated reads, the clean reads were processed for in silico analysis. Then, significance analysis of microarray (SAM) (version 4.0) was performed to select the mRNAs (Stanford University). Hierarchical cluster analysis was conducted using Gene Cluster 3.0 software (Stanford University).

### 2.6 Establishment of the rat IDD model

Three‐month‐old Sprague‐Dawley rats were used. All rats were of similar weight (approximately 380 g) to ensure that the tail discs at the position chosen for the experiment were of similar size so that, when they were punctured with a 20‐gauge needle, injuries of similar severity would be produced. Fifteen rats were randomly divided into three groups: the normal control group (NC; n = 5), the IDD model group (IDD; n = 5) and the IDD model with treatment with the Igfbp5 mRNA‐expressing lentivirus group (IDD + Igfbp5; n = 5). The lenti‐Igfbp5‐control and lenti‐Igfbp5 lentiviral particles were produced by triple transfection of 293T cells (Invitrogen) with the vectors pLVX‐Igfbp5‐control and pLVX‐Igfbp5, respectively, along with psPAX2 and pMD2.G. All the rats were anesthetized by the intraperitoneal injection of chloral hydrate (250 mg/kg). Subsequently, the IDD + Igfbp5 and IDD groups underwent annulus fibrosus needle puncture to induce surgical IDD. The injection of 10 µL lentivirus into the tail disc was performed at 7 days post‐IDD surgery. The IDD groups were injected with 10 µL lenti‐NC, while the experimental group was injected with lenti‐Igfbp5. The rats from each group were killed at 8 weeks post‐IDD surgery, and then the tails were dissected and processed for further histological evaluation.

### 2.7 Reverse transcription and quantitative real‐time PCR

To determine the mRNA levels in the NP samples, reverse transcription (RT) and quantitative real‐time PCR (qRT‐PCR) kits (Applied Biosystems) were used. The RT reactions were performed using the RevertAid RT Reverse Transcription Kit (Thermo Scientific™) in a total volume of 12 μL (65°C for 5 minutes, ice bath for 2 minutes, 42°C for 60 minutes, 70°C for 5 minutes and hold at −20°C).

qRT‐PCR was performed using a standard TaqMan PCR protocol. The 20‐μL PCRs included 4 μL diluted RT product, 20 μL TaqMan Universal PCR Master Mix with no AmpErase UNG, 1 μL TaqMan primers and 5 μL nuclease‐free water. The PCR primers were as follows: IGFBP5 (forward, ACCTGAGATGAGACAGGAGTC; reverse, GTAGAATCCTTTGCGGTCACAA), ERK1 (forward, ACTCACCTCTTCAGAACGAATTG; reverse, CCATCTTTGGAAGGTTCAGGTTG), ERK2 (forward, CCTGAACCTTCCAAAGATGGC; reverse, TTCACCAGGCAAGTCTCCTCA), Bax (forward, CCCGAGAGGTCTTTTTCCGAG; reverse, CCAGCCCATGATGGTTCTGAT), caspase‐3 (forward, CTGAACAGCTCCGAGGAAAC; reverse, TGGATATTCAGCCCTTTTGG), Bcl2 (forward, GGTGGGGTCATGTGTGTGG; reverse, CGGTTCAGGTACTCAGTCATCC) and GAPDH (forward, GGAGCGAGATCCCTCCAAAAT; reverse, GGCTGTTGTCATACTTCTCATGG). All reactions were performed in triplicate on a 7500 Real‐Time PCR system (Applied Biosystems) under the following conditions: 95°C for 10 minutes followed by 40 cycles of 95°C for 15 seconds and 60°C for 1 minute. Glyceraldehyde‐3‐phosphate dehydrogenase (GAPDH) was used as the internal control. The relative quantitative method was used to analyse the data, and the 2‐ΔΔCt method was applied to determine the relative expression of each target gene. The experiments were repeated three times independently.

### 2.8 Western blotting

According to standard methods, Western blot analysis was performed. Briefly, proteins were separated on 10% SDS‐PAGE gels and then transferred to PVDF membranes (Amersham). The membranes were blocked using 5% nonfat dried milk for 2 hours and then incubated for 12 hours with an anti‐IGFBP5 antibody (1:1000; Abcam), anti‐ERK antibody (1:1000; Abcam), anti‐pERK (phosphorylated ERK) antibody (1:500; Abcam), anti‐Bax antibody (1:1000; Abcam), anti‐caspase‐3 antibody (1:1000; Abcam), anti‐Bcl2 antibody (1:1000; Abcam) or anti‐GAPDH antibody (1:10 000; Abcam). After washing in TBST (10 mmol/L Tris, pH 8.0, 150 mmol/L NaCl and 0.1% Tween 20), the membranes were incubated for 2 hours with a goat anti‐rabbit antibody (1:5000; Abcam). Normalization was performed by blotting the same membranes with an antibody against GAPDH.

### 2.9 3‐(4,5‐Dimethylthiazol‐2‐yl)‐2,5‐diphenyltetrazolium bromide and colony formation assays

After the density of the transfected cells reached approximately 80%, the cells were washed twice with PBS and digested in 0.25% pancreatin to obtain single‐cell suspensions. After 7 and 14 days of culture in 24‐well plates, cell proliferation was assessed by a 3‐(4,5‐dimethylthiazol‐2‐yl)‐2,5‐diphenyltetrazolium bromide (MTT) assay. Briefly, the cells were cultured in 500 mL of MTT solution (250 mg in DMEM‐HG) at 37°C for 4 hours. During this incubation period, water‐insoluble formazan crystals were formed. Then, the formazan crystals were dissolved by adding 300 mL of dimethyl sulfoxide. The absorbance was measured at a wavelength of 570 nm using a Spectra MAX microplate reader (Molecular Devices).

For the colony formation assay, NP cells at the logarithmic growth phase in each transfected group were selected and washed once with PBS. Then, NP cells were plated in 2 mL of medium per well in six‐well plates. Following 10 days of incubation, the colonies were quantified after fixation with methyl alcohol and staining with haematoxylin. These experiments were performed three times.

### 2.10 Flow cytometry

Apoptosis was evaluated by staining cells with both Annexin V‐FITC and propidium iodide (PI) according to the manufacturer's instructions. Annexin V‐FITC was employed to quantitatively determine the percentage of cells undergoing apoptosis, which relies on the tendency of cells to lose membrane asymmetry in the early‐phase of apoptosis. In apoptotic cells, the membrane phospholipid phosphatidylserine is translocated from the inner leaflet of the plasma membrane to the outer leaflet, thereby exposing phosphatidylserine to the external environment. Cells that were positively stained with Annexin V‐FITC and unstained by PI were considered apoptotic. Cells that were positively stained for both Annexin V‐FITC and PI were considered necrotic.

### 2.11 Histological and immunohistochemical staining

Histological and immunohistochemical staining was performed on the intervertebral discs. Endogenous peroxidase was inactivated by incubating the sections with 0.3% H_2_O_2_ in PBS for 15 minutes, followed by a 30‐min block step with PBST/1% BSA. The sections were stained with haematoxylin and eosin (H&E) to show the structure of intervertebral disc. And then the sections were also incubated with 10 μg/mL rabbit anti‐collagen II antibody (Abcam) in PBST and 1% BSA at 4°C overnight. A goat anti‐rabbit IgG (H+L) (Abcam) was used as the secondary antibody for collagen II staining (2 µg/mL) for 1 hour at room temperature.

### 2.12 Immunofluorescent staining

Coverslips were placed into 24‐well plates in which NP cells were plated for 48 hours. The medium was removed, and the cells were washed twice with PBS and then fixed with 3.5% formaldehyde for 30 minutes at 37°C. The cells were rinsed with PBS three times, permeabilized with 0.1% Triton X‐100 in PBS for 20 minutes and blocked with 3% BSA and 0.05% Tween 20 in PBS for 30 minutes at room temperature. After blocking, the cells were incubated with 4 µg/mL rabbit polyclonal anti‐IGFBP5 (Abcam) overnight at 4°C. The cells were then treated with 2 µg/mL fluorescent goat anti‐rabbit secondary antibody (Abcam) for 1 hour at room temperature. The nuclei were stained with 4,6‐diamidino‐2‐phenylindole (DAPI).

### 2.13 Statistical analysis

All statistical analyses were performed using SPSS 17.0 software (SPSS Inc), and the graphs were generated using GraphPad Prism 5 Software (Graph Pad Software, Inc). Paired *t* tests, Student's *t* tests and Kruskal‐Wallis tests were used to analyse the mRNA and gene expression. ANOVAs were also performed to compare more than two groups. Pearson's correlation test was employed to evaluate the association between the expression of IGFBP5 mRNA and the disc degeneration grade in patients. *P* values (two‐tailed) <0.05 were considered to indicate statistical significance.

## 3 RESULTS

### 3.1 Identification of mRNAs differentially expressed in degenerative NP tissues

**Figure 1:**
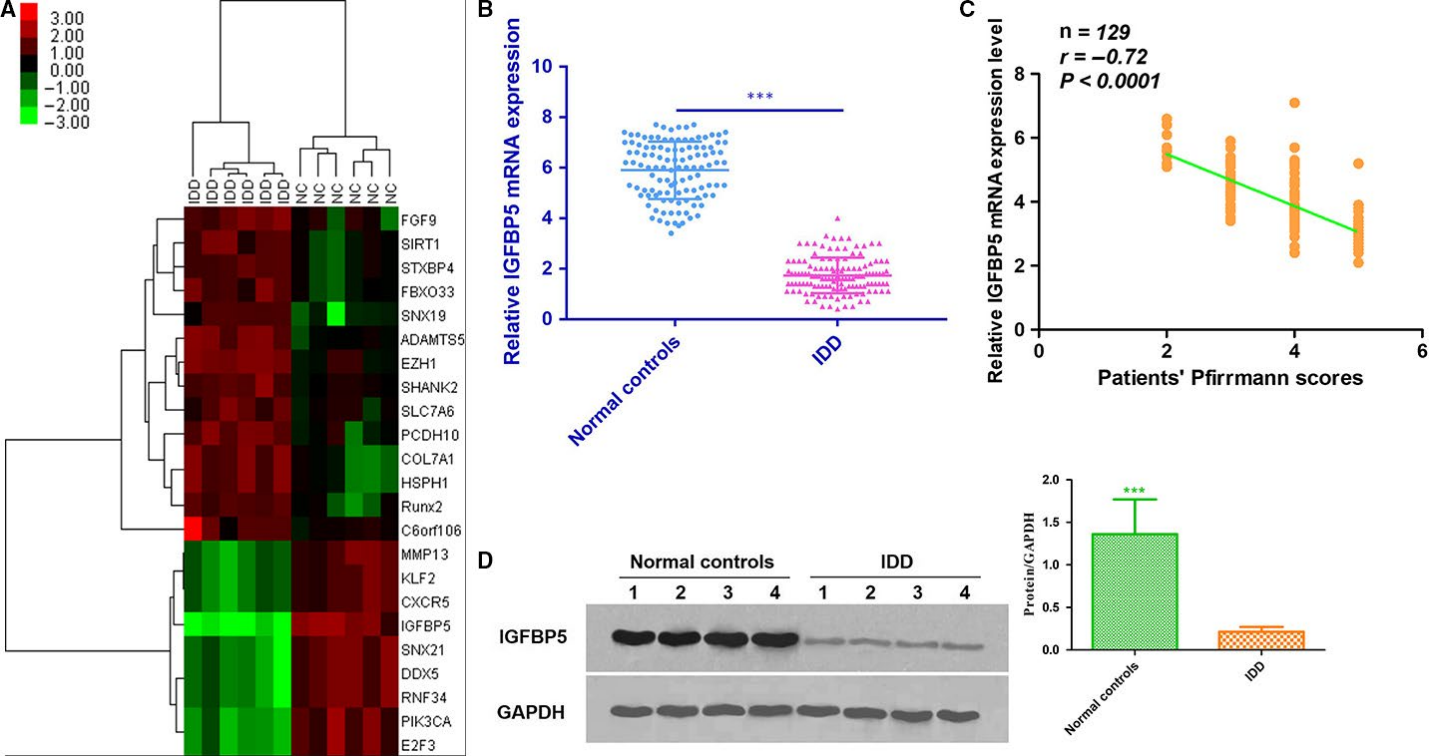
A, A heat map was generated by unsupervised clustering analyses with 23 significantly dysregulated mRNAs in patients with IDD. Hierarchical clustering was performed with average linkage and uncentered correlation. The mRNA expression profile effectively segregated patients with IDD from NCs. B, The expression level of IGFBP5 mRNA in NP cells was measured in 129 patients and 112 NCs (in the training and validation sets) using qRT‐PCR assays (***P < 0.001). C, The IGFBP5 mRNA expression levels were inversely correlated with the Pfirrmann scores (*r* = −0.72, *P* < 0.0001). D, The expression of IGFBP5 in degenerative NP tissues was significantly lower than that in normal NP samples, as assessed by Western blot analysis (*P* < 0.05). IDD, intervertebral disc degeneration; NCs, normal controls; NP, nucleus pulposus

The mRNA expression profiles were detected in 10 IDD tissues and 10 normal samples, and Solexa sequencing was performed to show the distinguishable mRNA expression patterns among the samples. On the basis of the mRNA expression profiling data, 89 mRNAs were found to be differentially expressed in IDD tissues, of which 41 mRNAs were up‐regulated and 48 mRNAs were down‐regulated. These differentially expressed serum miRNAs were chosen for further study only when they met the following criteria: (a) at least 20 copies of the mRNA were expressed; (b) a mean fold change >2.0 or <0.5 was determined; and (c) a *P* value <0.05 was determined. Based on these criteria, 23 mRNAs, of which 14 mRNAs were up‐regulated and 9 mRNAs were down‐regulated in patients compared with controls, were chosen for further validation (Figure 1A).

A qRT‐PCR assay was used to confirm the expression of the candidate mRNAs. For the training set, the mRNAs were measured in a separate set of samples from 10 patients and 10 controls from the previous step. Only mRNAs with a mean fold change >2.0 or <0.5 and a *P* value <0.01 were selected for further analysis. On the basis of the abovementioned criteria, SIRT1, PCDH10, IGFBP5 and PIK3CA were observed to be significantly different in patients compared with controls (Table 1). For the validation set, the concentrations of SIRT1, PCDH10, IGFBP5 and PIK3CA were measured by qRT‐PCR in a larger cohort comprising 129 patients and 112 controls. The IGFBP5 expression pattern in the validation set was consistent with that found in the training set. Compared with the controls, the level of IGFBP5 mRNA was significantly lower in patients (Table 1). Thus, we focused on IGFBP5 mRNA for further study.

**Table 1:** Differentially expressed mRNAs in IDD patients and normal controls in both the training set and the validation set

mRNA	Training set		Validation set	
	Fold change	*P* value	Fold change	*P* value
Up‐regulated				
FGF9	4.1	0.23	‐	‐
**SIRT1**	**7.4**	**0.006** **	**7**	**0.15**
STXBP4	4.1	0.32	‐	‐
FBXO33	8.9	0.34	‐	‐
SNX19	7.5	0.09	‐	‐
ADAMTS5	6.1	0.34	‐	‐
EZH1	7.3	0.45	‐	‐
SHANK2	6.2	0.38	‐	‐
SLC7A6	7.6	0.27	‐	‐
**PCDH10**	**6.6**	**0.004** **	**6.2**	**0.32**
COL7A1	8.2	0.78	‐	‐
HSPH1	7.1	0.29	‐	‐
Rux2	4.7	0.13	‐	‐
C6orf106	6.2	0.07	‐	‐
Down‐regulated				
MMP13	0.19	0.14	‐	‐
KLF2	0.18	0.26	‐	‐
CXCR5	0.31	0.87	‐	‐
**IGFBP5**	**0.02**	**0.002** **	**0.031**	**0.005** **
SNX21	0.11	0.43	‐	‐
DDX5	0.37	0.35	‐	‐
RNF34	0.21	0.56	‐	‐
**PIK3CA**	**0.18**	**0.006** **	**0.11**	**0.32**
E2F3	0.12	0.43	‐	‐

IDD group vs. normal group (in bold).

** *P* < 0.01.

### 3.2 Down‐regulation of IGFBP5 expression is associated with IDD

To determine the expression level of IGFBP5 mRNA in degenerative NP tissues, we measured the level of IGFBP5 mRNA in 129 patients and 112 controls. The results demonstrated that the level of IGFBP5 mRNA was down‐regulated in degenerative NP tissues when compared with control tissues (Figure 1B). In addition, the expression of IGFBP5 mRNA was negatively correlated with the disc degeneration grade (*r* = −0.72, *P* < 0.0001) (Figure 1C). Moreover, the expression of IGFBP5 was significantly lower in degenerative NP tissues than in normal NP samples according to Western blot analysis (*P* < 0.05, Figure 1D).

### 3.3 IGFBP5 promotes NP cell proliferation and inhibits apoptosis in vitro

**Figure 2:**
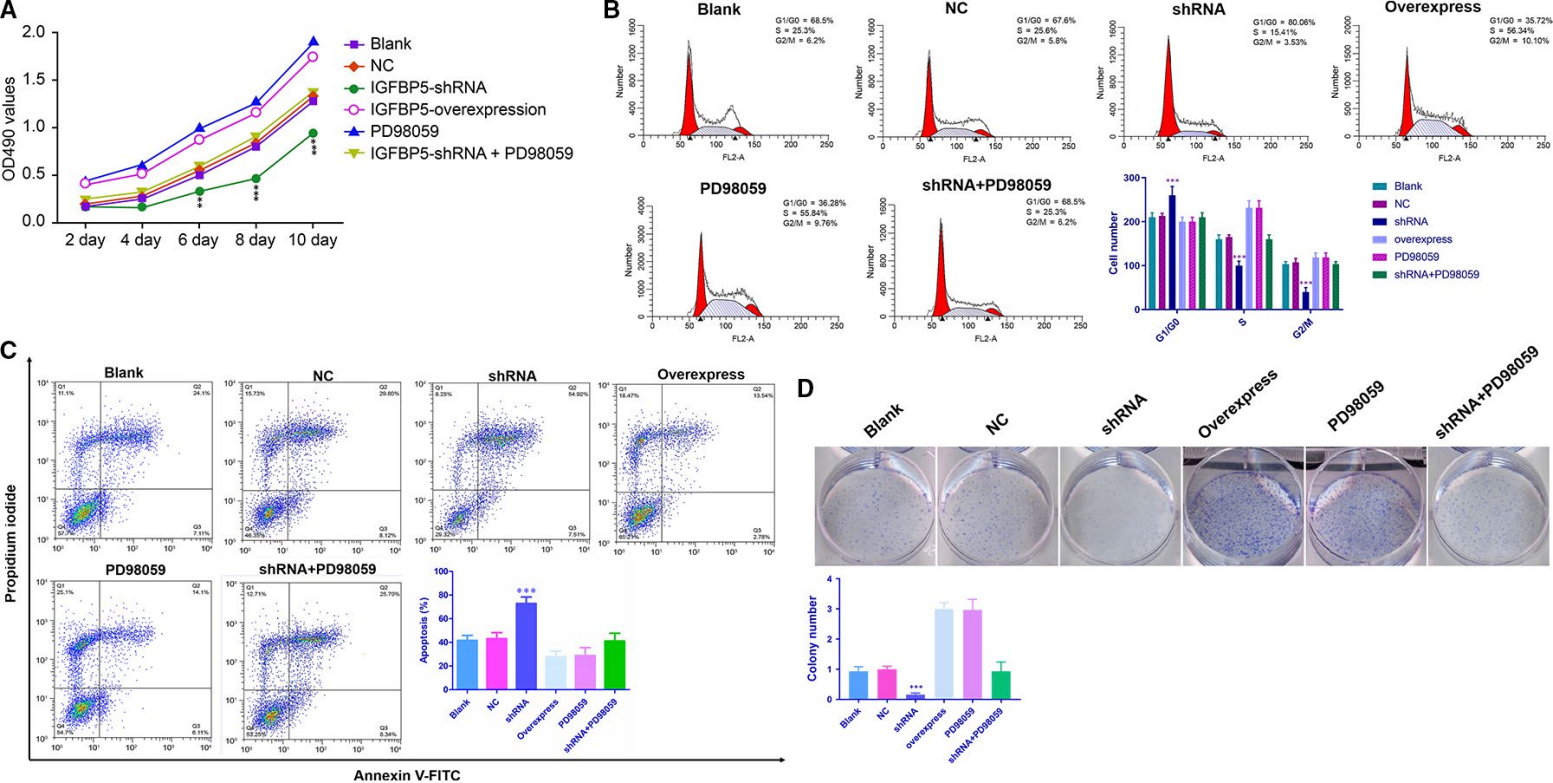
Analysis of cell proliferation and apoptosis. A, An MTT assay showed that IGFBP5 overexpression increased cellular proliferation at day 10; **P < 0.01 and ***P < 0.001 compared with the normal control. B‐D, Flow cytometry and colony formation assays demonstrated that IGFBP5 overexpression could effectively promote NP cell proliferation and inhibit NP cell apoptosis. In contrast, IGFBP5‐shRNA could suppress NP cell proliferation and promote NP cell apoptosis; ***P < 0.001 compared with the normal control. NP, nucleus pulposus

At 10 days following IGFBP5‐overexpressing plasmid transfection, cell proliferation was significantly higher when compared to the transfection of IGFBP5‐shRNA (*P* < 0.01) (Figure 2A). The PI staining results revealed that, compared with those in the NC group, the percentages of cells in the G1 phase in the IGFBP5‐overexpressing and PD98059‐treated groups were significantly decreased, while the percentages of cells in the S phase were significantly increased (all *P* < 0.001, Figure 2B). In contrast, IGFBP5 silencing led to cell growth arrest at the G1 phase, and the number of cells in the S phase decreased, indicating the suppression of NP cell proliferation (all *P*s < 0.001, Figure 2B). With respect to NP cell apoptosis, the Annexin V apoptosis assay demonstrated that IGFBP5‐overexpressing plasmid transfection and PD98059 treatment significantly decreased the number of apoptotic NP cells compared with IGFBP5‐shRNA transfection (*P* < 0.001, Figure 2C). This effect was further confirmed by a colony formation assay (Figure 2D). Moreover, there were no differences between the blank, NC and shRNA + PD98059 groups in NP cell proliferation and apoptosis (Figure 2A‐D).

### 3.4 IGFBP5 exerts its effects by inhibiting the ERK signalling pathway

**Figure 3:**
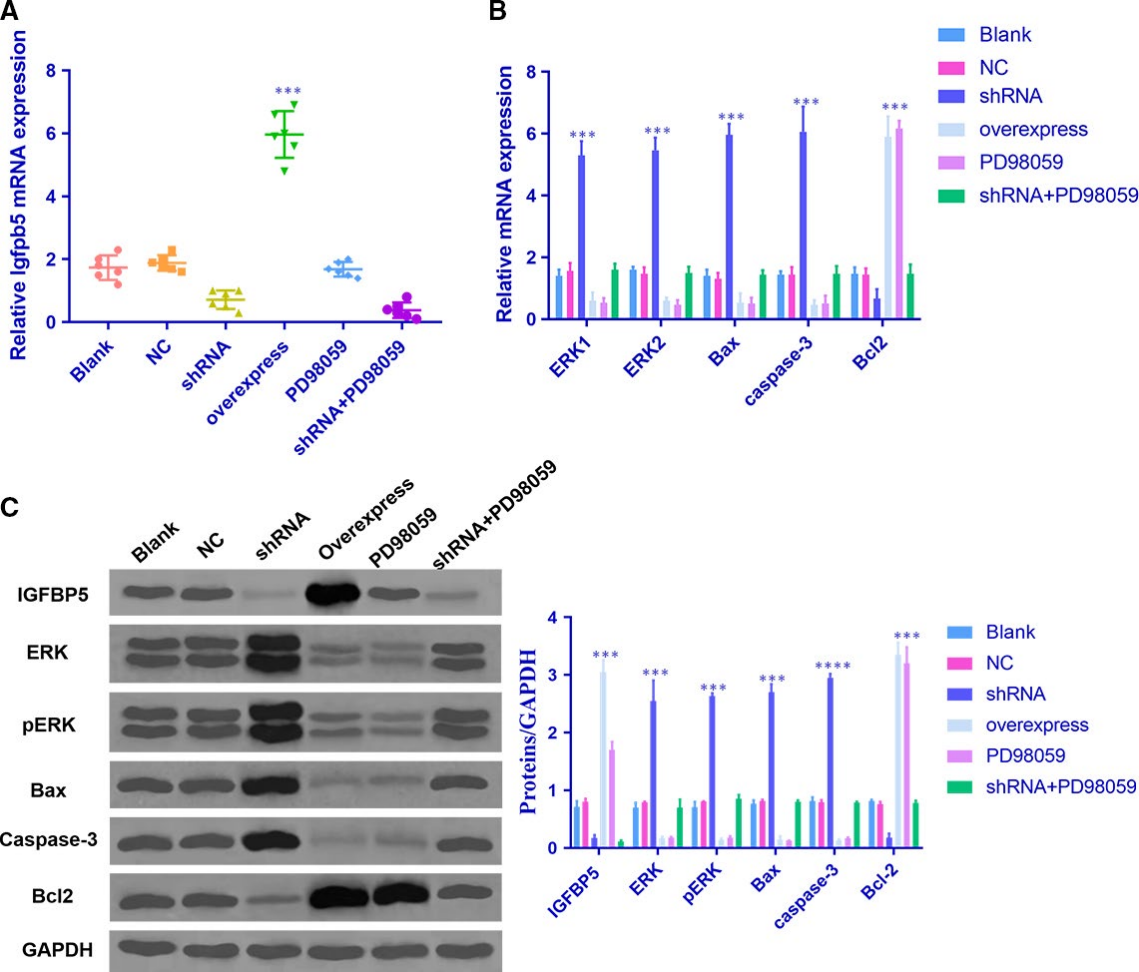
IGFBP5 exerts its effects by inhibiting the ERK signalling pathway. A, The NP cells in each group were transfected and showed high transfection efficiency; ***P < 0.001 compared with the normal control. B and C, The overexpression of IGFBP5 inhibited ERK signalling pathways in NP cells. qRT‐PCR and Western blot analyses demonstrated that IGFBP5 overexpression led to decreases in ERK, pERK, Bax and caspase‐3; ***P < 0.001 compared with the normal control. In NP cells treated with IGFBP5‐shRNA, the expression levels of ERK, pERK, Bax and caspase‐3 were significantly increased, while the level of Bcl2 was decreased; ***P < 0.001 compared with the normal control. The values are presented as the mean ± SD. NP, nucleus pulposus

The NP cells in each group were transfected and showed high transfection efficiency (Figure 3A). To explore whether IGFBP5 exerts its effects through the ERK signalling pathway, which contributes to NP cell proliferation and survival, we performed qRT‐PCR and Western blot analyses to examine the levels of several genes and related proteins, including ERK, pERK, Bax, caspase‐3 and Bcl2. The expression of ERK, pERK, Bax and caspase‐3 was decreased, while the level of Bcl2 was increased in NP cells that stably overexpressed IGFBP5 mRNA (Figure 3B,C). Additionally, the effect of IGFBP5 mRNA overexpression on NP cells was similar to that induced by the ERK inhibitor PD98059. In contrast, the expression of ERK, pERK, Bax and caspase‐3 was significantly up‐regulated, while the level of Bcl2 was down‐regulated following transfection with IGFBP5‐shRNA (Figure 3B,C). No differences in the expression levels of these genes and related proteins were observed between the IGFBP5‐shRNA + PD98059, blank and NC groups (all *P*s > 0.05). These data indicate that IGFBP5 mRNA inhibits the ERK signalling pathway in IDD.

### 3.5 IGFBP5 inhibited disc degeneration in the IDD model

**Figure 4:**
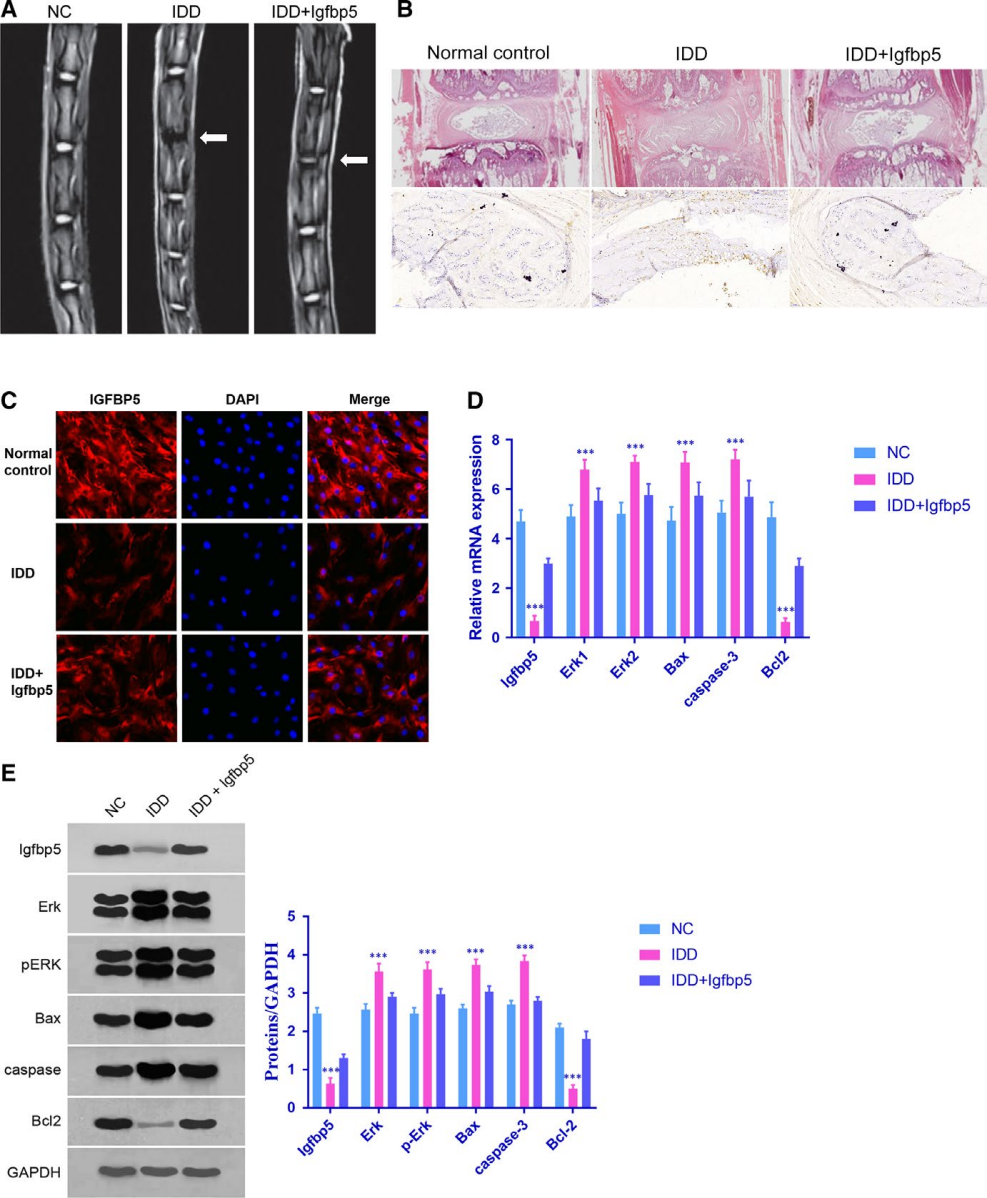
Effect of IGFBP5 in IDD rats. A and B, Severe intervertebral disc degeneration was induced in the rats in the IDD group. Igfbp5 mRNA expression treatment abrogated the effects on the intervertebral disc tissues. C and D, The expression level of IGFBP5 in NP cells was detected by immunofluorescence staining and qRT‐PCR; ***P < 0.001 compared with the normal control. D and E, The mRNA expression levels of Erk1/2, Bax, caspase‐3 and Bcl2 were measured by qRT‐PCR. The protein expression levels of ERK, pERK, Bax, caspase‐3 and Bcl2 were measured by Western blotting; ***P < 0.001 compared with the normal control. Data are shown as the mean ± SD of five rats in each group. NC = normal control group, IDD = IDD model group, IDD + Igfbp5=IDD model rats treated with an Igfbp5 mRNA‐expressing lentivirus

To investigate the role of IGFBP5 in vivo, a rat model of IDD was generated. As shown in Figure 4A,B, the progression of IDD in the IDD + Igfbp5 group was inhibited compared with that in the IDD group. Moreover, the IGFBP5 level in NP cells was lower in IDD rats than in IDD + Igfbp5 rats (*P* < 0.0001), which was confirmed by the immunofluorescence staining and qRT‐PCR results (Figure 4C,D). The mRNA expression levels of Erk1/2, Bax, caspase‐3 and Bcl2 were measured by qRT‐PCR (Figure 4D). The protein expression levels of ERK, pERK, Bax, caspase‐3 and Bcl2 were measured by Western blotting (Figure 4E). Compared with those in the IDD rats, the ERK, pERK, Bax and caspase‐3 expression levels in the IDD + Igfbp5 rats were lower (*P* < 0.0001). In contrast, the level of Bcl2 was higher in the IDD + Igfbp5 rats than in the IDD rats (*P* < 0.0001).

## 4 DISCUSSION

In the current study, we first performed state‐of‐the‐art next‐generation sequencing (NGS) in degenerative NP tissues and normal disc tissues to identify IDD‐specific mRNAs. Using the Illumina HiSeq 2500 platform and subsequent qRT‐PCR, IGFBP5 mRNA was found to be significantly down‐regulated in patients compared with the corresponding controls and was thus selected for further analysis. Moreover, the IGFBP5 expression level was significantly lower in degenerative NP tissues and was significantly correlated with the disc degeneration grade. To further investigate the role of IGFBP5 in the pathogenesis of IDD, we performed functional analysis of IGFBP5 to investigate the relationship between IGFBP5 and NP cell proliferation and apoptosis. In our study, NP cell proliferation was significantly increased by the overexpression of IGFBP5, whereas it was significantly inhibited by decreased expression of IGFBP5 induced by transfection with IGFBP5‐shRNA. In addition, the overexpression of IGFBP5 led to an increase in cell growth and in the percentage of cells in the S phase of the cell cycle and led to a decrease in the cell apoptosis rate and the percentage of cells in the G1 phase. Contrasting results were observed following transfection with IGFBP5‐shRNA. These findings suggest that increased NP cell proliferation induced by the overexpression of IGFBP5 may be one of the possible mechanisms involved in IDD development.

The regulation of cell proliferation and apoptosis is a complex process and is associated with several signalling pathways.^16^, ^17^, ^18^, ^19^, ^20^ Extracellular signal‐regulated kinase (ERK), which is involved in the Ras‐Raf‐MEK‐ERK pathway, plays an important role in the regulation of cell differentiation.^21^, ^22^ It is activated through a sequential phosphorylation cascade that amplifies and transduces signals from the cell membrane to the nucleus^23^, ^24^, ^25^, ^26^ and is inhibited by PD98059. PD98059 is a highly selective in vitro inhibitor of MEK1 activation by binding to inactive forms of MEK1. Phosphorylated MEK1 can further activate ERK by phosphorylation and evoke its downstream protein kinase cascade.^27^ Therefore, PD98059 down‐regulates pERK level by inhibiting the phosphorylation of MEK1. As revealed in our results, the down‐regulation in ERK occurred in the group treated with PD98059. We assumed that PD98059 inhibits the activation of MEK1 and then may down‐regulate the level of ERK, with parallel to the down‐regulation of pERK level.

Both in vitro and in vivo data have indicated that the proliferation and apoptosis of human NP cells are regulated by exogenous and autocrine growth factors that mainly act via the ERK pathway.^13^, ^18^, ^28^ To determine the potential link between IGFBP5 and the ERK signalling pathway, IGFBP5 mRNA was overexpressed in NP cells by transfection, and the results showed that the expression of both ERK and phosphorylated ERK was attenuated in IGFBP5‐overexpressing cells, which was similar to PD98059 treatment, indicating that IGFBP5 inhibits cell apoptosis by regulating the ERK pathway. Conversely, the phosphorylation of these kinases was enhanced in IGFBP5 knockdown cells, which was confirmed by Western blot and qRT‐PCR analyses. In addition, increased activity of ERK pathway‐associated proteins such as Bax^29^, ^30^, ^31^ and caspase‐3^32^, ^33^ and the down‐regulation of antiapoptotic proteins such as Bcl2^34^, ^35^, ^36^ is associated with the up‐regulation of apoptosis. The results obtained from the qRT‐PCR and Western blot analyses indicated that the Bax and caspase‐3 levels were significantly increased, while the level of Bcl2 was significantly decreased in the IGFBP5‐shRNA group. All the above findings demonstrate that the silencing of IGFBP5 induced the apoptosis of NP cells by activating the ERK pathway, which further up‐regulated Bax and caspase‐3 and down‐regulated Bcl2.

Another interesting aspect of our study is that we demonstrated the role of IGFBP5 in IDD for the first time using a rat model. Our data revealed that the IGFBP5 mRNA level in NP cells was lower in IDD rats when compared with IDD + Igfbp5 rats (*P* < 0.0001). Additionally, the Bcl2 expression level was relatively high in IDD + Igfbp5 rats, while the levels of ERK, pERK, Bax and caspase‐3 were higher in the IDD rats. Collectively, our findings provide novel mechanistic insights that have been previously unrecognized and that highlight that the suppression of IGFBP5 is an early phenomenon in IDD that may trigger the initiation of IDD.

Overall, this study has resulted in the discovery and validation of IDD‐specific mRNA transcriptome profiles generated by NGS. We found that IGFBP5 was down‐regulated in human degenerative NP tissues and that its level was associated with the disc degeneration grade. In addition, the inhibitory effects of down‐regulated IGFBP5 are, in part, mediated through the ERK signalling pathway. By the use of in vivo and in vitro studies, functional characterization of the role of IGFBP5 revealed that the loss of its expression occurs during IDD pathogenesis. Therefore, strategies to maintain the expression or to prevent the repression of IGFBP5 have the potential to serve as possible therapeutic and/or preventive approaches for degenerative disc disease.

## CONFLICT OF INTEREST

The authors declare no conflicts of interest.

## AUTHOR'S CONTRIBUTION

All authors were involved in drafting the article or revising it critically and providing important intellectual content, and all authors approved the final version. Dr Yaming Li had full access to all the data in the study and takes responsibility for the integrity of the data and the accuracy of the data analysis. Weibing Zhang and Nu Zhang were involved in the study conception and design. Zhonghui Chen, Yan Zhou, Geliang Hu, Mingdi Xue and Junhua Liu were responsible for the acquisition of the data. Zhonghui Chen, Yan Zhou and Geliang Hu analysed and interpreted the data. Zhonghui Chen prepared the manuscript.

## Data Availability

The datasets generated during and/or analysed during the current study are available from the corresponding author on reasonable request.
