# Correction to Downregulation of insulin‐like growth factor binding protein 5 is involved in intervertebral disc degeneration via the ERK signalling pathway

**DOI:** 10.1111/jcmm.17876

**Published:** 2023-08-23

In Zhonghui Chen et al.,^1^ the published article contains errors in Figure 4A. The correct figure is shown below. The authors confirm that all results and conclusions of this article remain unchanged.

**FIGURE 4:**
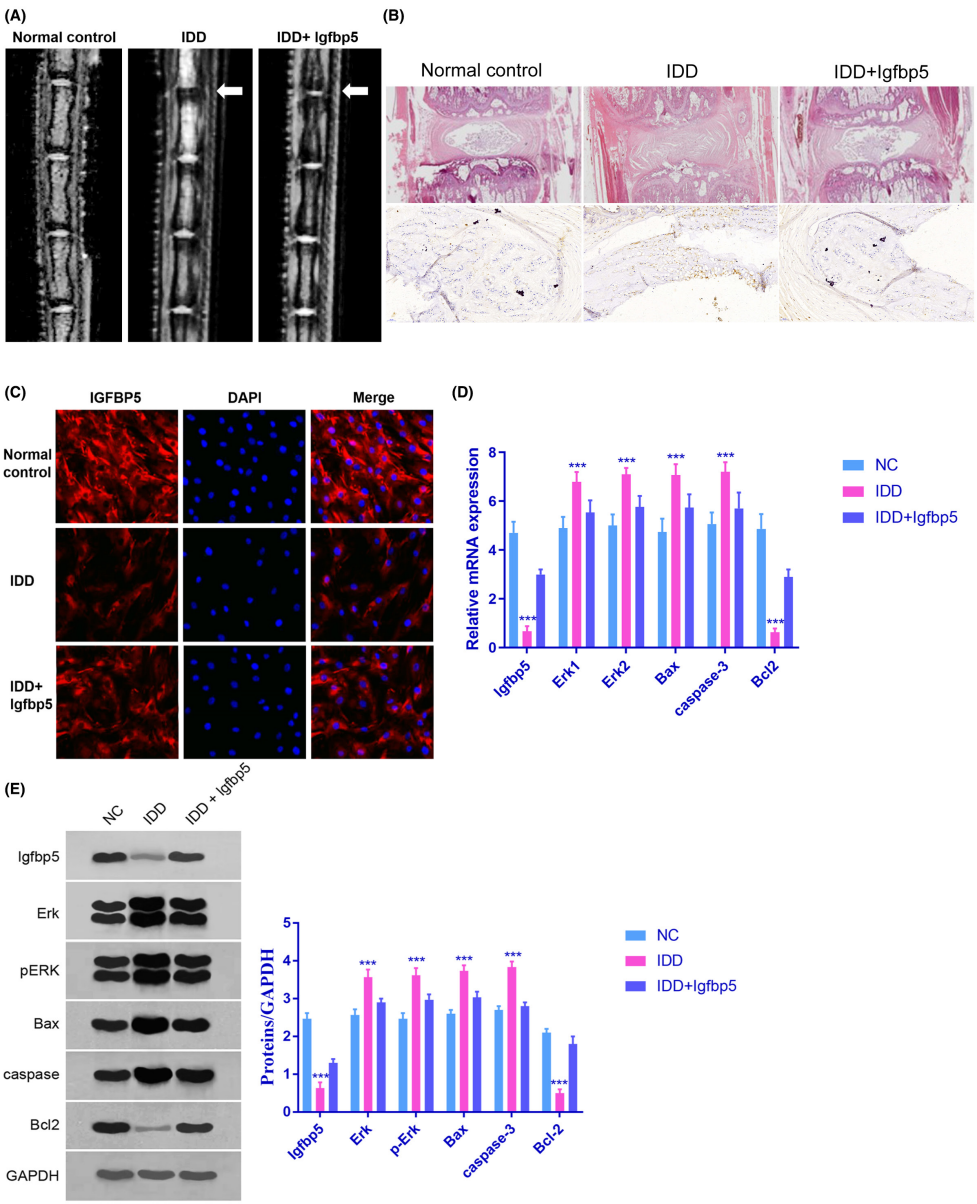
Effect of IGFBP5 in IDD rats. (A and B) Severe intervertebral disc degeneration was induced in the rats in the IDD group. Igfbp5 mRNA expression treatment abrogated the effects on the intervertebral disc tissues. (C and D) The expression level of IGFBP5 in NP cells was detected by immunofluorescence staining and qRT‐PCR; ***p < 0.001 compared with the normal control. (D and E) The mRNA expression levels of Erk1/2, Bax, caspase‐3 and Bcl2 were measured by qRT‐PCR. The protein expression levels of ERK, pERK, Bax, caspase‐3 and Bcl2 were measured by western blotting; ***p < 0.001 compared with the normal control. Data are shown as the mean ± SD of five rats in each group. NC = normal control group, IDD = IDD model group, IDD + Igfbp5 = IDD model rats treated with an Igfbp5 mRNA‐expressing lentivirus.
